# A normalized contrast‐encoding model exhibits bright/dark asymmetries similar to early visual neurons

**DOI:** 10.14814/phy2.12746

**Published:** 2016-04-06

**Authors:** Emily A. Cooper

**Affiliations:** ^1^ Department of Psychological & Brain SciencesDartmouth CollegeHanoverNew Hampshire

**Keywords:** Lateral geniculate nucleus, ON/OFF pathways, retinal ganglion cells, visual contrast

## Abstract

Biological sensory systems share a number of organizing principles. One such principle is the formation of parallel streams. In the visual system, information about bright and dark features is largely conveyed via two separate streams: the ON and OFF pathways. While brightness and darkness can be considered symmetric and opposite forms of visual contrast, the response properties of cells in the ON and OFF pathways are decidedly asymmetric. Here, we ask whether a simple contrast‐encoding model predicts asymmetries for brights and darks that are similar to the asymmetries found in the ON and OFF pathways. Importantly, this model does not include any explicit differences in how the visual system represents brights and darks, but it does include a common normalization mechanism. The phenomena captured by the model include (1) nonlinear contrast response functions, (2) greater nonlinearities in the responses to darks, and (3) larger responses to dark contrasts. We report a direct, quantitative comparison between these model predictions and previously published electrophysiological measurements from the retina and thalamus (guinea pig and cat, respectively). This work suggests that the simple computation of visual contrast may account for a range of early visual processing nonlinearities. Assessing explicit models of sensory representations is essential for understanding which features of neuronal activity these models can and cannot predict, and for investigating how early computations may reverberate through the sensory pathways.

## Introduction

Visual contrast – a change in the luminance or wavelength of light relative to a local average – is a primary signal transmitted by the retina. This is likely because contrast provides a useful and efficient representation of behaviorally relevant information (Land and McCann 1971; Laughlin 1981; Shapley and Enroth‐Cugell 1984; Brady and Field 2000). The retina conveys contrast information to the brain via two segregated pathways devoted to brightness (ON) and darkness (OFF) (Hartline 1938; Kuffler 1953; Werblin and Dowling 1969; Schiller et al. 1986; Westheimer 2007). Intuitively, neurons in the ON and OFF pathways might be expected to respond similarly to stimuli of equal and opposite contrast. But in fact, the contrast response functions of ON and OFF neurons have systematic differences (Chichilnisky and Kalmar 2002; Zaghloul et al. 2003; Liang and Freed 2010; Kremkow et al. 2014; Jiang et al. 2015). ON/OFF asymmetries also extend to neuronal spatial receptive fields and temporal properties (Dacey and Petersen 1992; Chichilnisky and Kalmar 2002; Pandarinath et al. 2010; Ratliff et al. 2010; Jin et al. 2011; Nichols et al. 2013).

But the computational role that these asymmetries play in early visual processing is an open question. In this report, we examine whether differences in ON and OFF contrast response functions necessarily reflect differences in their underlying representations of visual information. This work is motivated by two main observations: (1) some ON/OFF asymmetries may arise at the level of the photoreceptor, prior to the point at which bright and dark information become segregated (Angueyra and Rieke 2013; Kremkow et al. 2014; Carandini 2016), and (2) several simple models proposed for physiologically plausible contrast encoding contain nonlinearities, but these models have not been directly compared to the nonlinearities of ON and OFF neurons (Peli 1990; Tadmor and Tolhurst 2000; Ratliff et al. 2010; Haun and Peli 2013).

Here, we define an explicit computational model of contrast encoding and directly compare the predicted responses from this model to a range of previously reported electrophysiological measurements from different species (guinea pig and cat) and cell types (retinal ganglion cells [RGCs] and lateral geniculate cells). While the exact details of early visual response characteristics vary between species (Chichilnisky and Kalmar 2002; Zaghloul et al. 2003; Liang and Freed 2010; Nichols et al. 2013), we ask whether a simple model can capture common features across species. Surprisingly, we find that many differences in the response characteristics of ON and OFF neurons can be recreated using a single, basic contrast‐encoding model. This is because asymmetries naturally result from a fundamental neural computation – divisive normalization (Carandini and Heeger 2012).

## Materials and Methods

### Model

**Figure 1:**
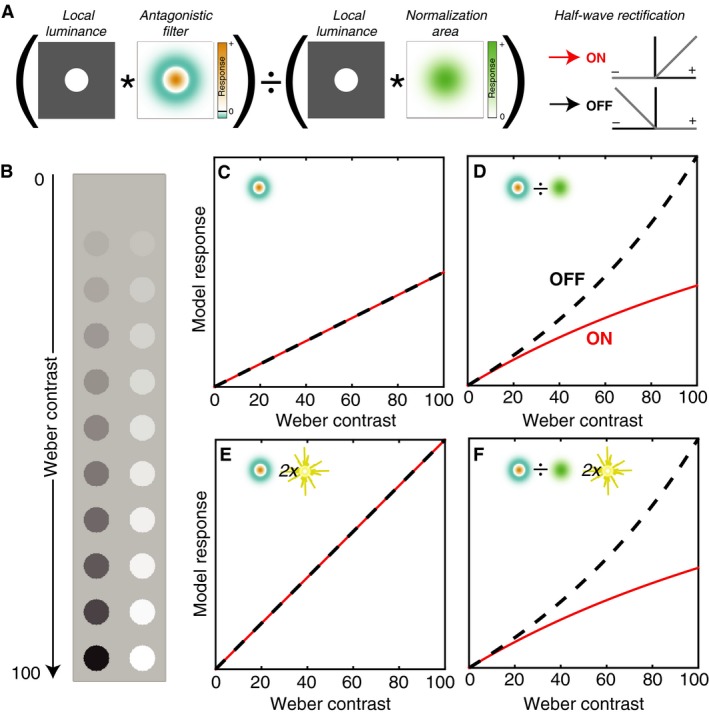
Measuring luminance contrast. (A) In the model, an image is convolved (*) with both an antagonistic and a low‐pass filter. The antagonistic filter output is divided (normalized) by the low‐pass output. Half‐wave rectification is used to segregate bright (ON) and dark (OFF) responses without additional nonlinearities. (B) A disk in equal steps of Weber contrast from 0 to 100%, either decremental/dark (left) incremental/bright (right). (C, D) Predicted ON and OFF contrast response functions for the antagonistic stage alone (C) and the full model (D). (E, F) Response functions are shown as in (C, D), except the stimulus background level was increased by a factor of two. The ordinate scales are matched for C/E and D/F.

We employ a generic, yet physiologically plausible contrast‐encoding model derived from previous definitions of visual contrast (Fig. 1A) (Peli 1990; Tadmor and Tolhurst 2000; Bex and Makous 2002; Ratliff et al. 2010; Haun and Peli 2013). The model consists of a linear *antagonistic* step that detects spatial changes in luminance, and a nonlinear *normalization* step in which the antagonistic output is divided by the local average luminance (i.e., a 2D divisively normalized difference of Gaussians (DoG)).

Thus, the model response – *r*, a scalar value – to a particular stimulus is:

(1) $$r=\frac{f\left(x,y\right)*(g\left(x,y;\sigma_{c}\right)-g\left(x,y;\sigma_{s}\right))}{f\left(x,y\right)*g\left(x,y;\sigma_{n}\right)},$$

where the luminance image, *f*(*x*,*y*), is defined over a 2D lattice of spatial positions with origin (0,0) at the image center, and where * is the convolution operator. Each convolution filter, *g*(*x*,*y*;*σ*), is a 2D Gaussian of the form:

(2) $$g\left(x,y;\sigma \right)=\frac{1}{2\pi \sigma^{2}}e{}^{\frac{-(x^{2}+y^{2})}{2\sigma^{2}}}.$$

Note that for the stimuli described here, the luminance image and the filter are the same size, so the convolution is computed at a single point in the center of the image, and thus the response is a scalar value.

Three different standard deviations (*σ*_*c*_, *σ*_*s*_, and *σ*_*n*_) determine the relative shapes/sizes of the antagonistic (center, surround) and normalization steps, respectively. Modifying these standard deviations changes the spatial extent over which contrast is computed. To create a family of filters with different shapes and sizes, *σ*_*c*_ was fixed in arbitrary units and *σ*_*s*_ and *σ*_*n*_ were varied by scale factors. *σ*_*s*_ ranged from 1.25× to 6× *σ*_*c*_ (1.25, 1.5, 2, 3, 4, 6); *σ*_*n*_ ranged from 1× to 6× *σ*_*c*_ (1, 1.25, 1.5, 2, 3, 4, 6). Between these two parameters, 42 unique model units were analyzed.

While we do not propose this as a literal model of early visual processing, the DoG filter captures the center‐surround antagonism and the spatial properties of early visual neuron receptive fields (both RGCs and cells in the lateral geniculate nucleus [LGN]) (Enroth‐Cugell and Robson 1966; Derrington and Lennie 1984; Croner and Kaplan 1995). The range of surround/center ratios explored is consistent with those measured in cat RGCs (Linsenmeier et al. 1982). The inclusion of normalization is reflective of the response adaptation that results from retinal gain control mechanisms (Shapley and Enroth‐Cugell 1984). Note, however, that this model considers only spatial properties of contrast‐encoding, whereas early visual neurons also encode temporal contrast (e.g., Derrington and Lennie 1984; Lee et al. 1989).

After filtering a stimulus, we modeled ON/OFF parallelization by performing half‐wave rectification that preserved only the positive (ON) or negative (OFF) outputs. Importantly, we did not apply any additional nonlinearity after this rectification, because our goal was to examine the predicted output in the absence of any explicit bright/dark encoding asymmetries. Thus, the modeling steps leading up to predicting ON and OFF responses were completely symmetric.

### Stimuli

To measure the model contrast response functions, stimuli consisted of images of uniform disks on a uniform background. The background level was either 50 or 100 (arbitrary units), and the disks ranged in Weber contrast from −100 to 100%. Weber contrast is defined as 100(*d *− *b*)/*b*, where *d* and *b* are the luminance of the disk and background, respectively. The disk diameter was 4× the standard deviation of the model central Gaussian. For the luminance response analysis (Fig. 4), stimuli instead consisted of squares on one of three background intensities (2, 61, or 120), so as to match the Kremkow et al. (2014) stimulus values in cd/m^2^. The width of the squares was also 4× the standard deviation of the central Gaussian.

### Analysis

For the comparison with guinea pig RGC responses (Zaghloul et al. 2003), nonlinearity indices were calculated for the model units by taking the log of the ratio of the contrast response function slope at 50% contrast and 5% contrast. Slopes were estimated using finite differences. This was similar to the nonlinearity indices in Zaghloul et al. (2003) and other studies (Chichilnisky and Kalmar 2002; Kastner and Baccus 2013). To ensure that the results were not specific to this particular index, this calculation was repeated for slopes measured at contrasts ranging from 20 to 90%.

For the comparison with cat LGN cell responses (Kremkow et al. 2014), values of luminance half‐response saturation (L50) were computed by finding the target luminance that resulted in a response that was half as large as the response at the largest luminance contrast that was presented for a given stimulus (Rmax). As in the previous report (Kremkow et al. 2014), these values were then converted to proportions.

Note that in both studies to which the model predictions were compared (Zaghloul et al. 2003; Kremkow et al. 2014), the response functions reflect the initial neuronal response to a change in contrast. In Kremkow et al. (2014), this was the number of spikes recorded in the 100 ms during which the stimulus was flashed on a uniform background. In Zaghloul et al. (2003), this was the instantaneous nonlinearity component of a model fit to neuronal responses to white noise stimulation (in terms of spikes per second). Since the Weber contrast of the Zaghloul et al. (2003) stimulus was not defined, comparisons to these data are limited to the nonlinearity indices described above.

## Results

### Different contrast response functions for brights and darks

The contrast response function of a neuron is characterized by plotting the average spike rate as a function of the contrast of a stimulus presented within the receptive field (Fig. 1B). Several different studies have produced similar conclusions about the contrast responses of precortical visual neurons to spots of bright and dark contrast. *First*, the responses do not vary linearly as a function of contrast (Burkhardt et al. 1998; Chichilnisky and Kalmar 2002; Zaghloul et al. 2003; Kremkow et al. 2014). *Second*, the nonlinearity is stronger for OFF cells than for ON cells over the same range of contrast magnitudes (Chichilnisky and Kalmar 2002; Zaghloul et al. 2003; Kastner and Baccus 2013). *Third*, the response of OFF cells at high contrasts is greater than ON cells (Zaghloul et al. 2003; Kremkow et al. 2014).

Each of these features is captured in the contrast response functions of the model. Response functions from an example model unit are shown in Figure 1C–F (*σ*_*s*_ = *σ*_*n*_ = 2*σ*_*c*_). For ease of comparison, Weber contrast is plotted in terms of absolute value for bright (ON) and dark (OFF) contrasts. Model ON responses are shown as solid red lines and model OFF responses are shown as dashed black lines. While the antagonistic step in isolation produces linear and symmetric ON/OFF response functions (Fig. 1C), the inclusion of normalization causes the model responses to become asymmetric and nonlinear (Fig. 1D). For this example unit, the response to darks has an accelerating nonlinearity, and the response to brights is decelerating. When the background illumination level is increased by a factor of two, the response function of the antagonistic stage alone also increases by a factor of two (Fig. 1C and E). However, the response function of the full model is unchanged (Fig. 1D and F).

Thus, the inclusion of normalization in the full model results in contrast constancy *within* a polarity at the cost of contrast constancy *between* polarities. Within a polarity (bright or dark), contrast responses are the same regardless of the overall illumination level – but for a given contrast level, the bright and dark responses differ from each other. This asymmetry arises because of differences between the values that go into the normalization (eq. 1). For dark points, the value of the normalization area decreases as the spot gets darker, and the decreasing values in the divisor result in a boosting of the OFF response. For bright points, the reverse is true: as the spot gets brighter, the divisor gets larger, and the ON response begins to saturate. This effect is sufficient to create asymmetric contrast response functions. Note, however, that this analysis necessarily excludes the modeling of ON responses to dark contrasts and OFF responses to bright contrasts, which have additional asymmetries (Chichilnisky and Kalmar 2002).

**Figure 2:**
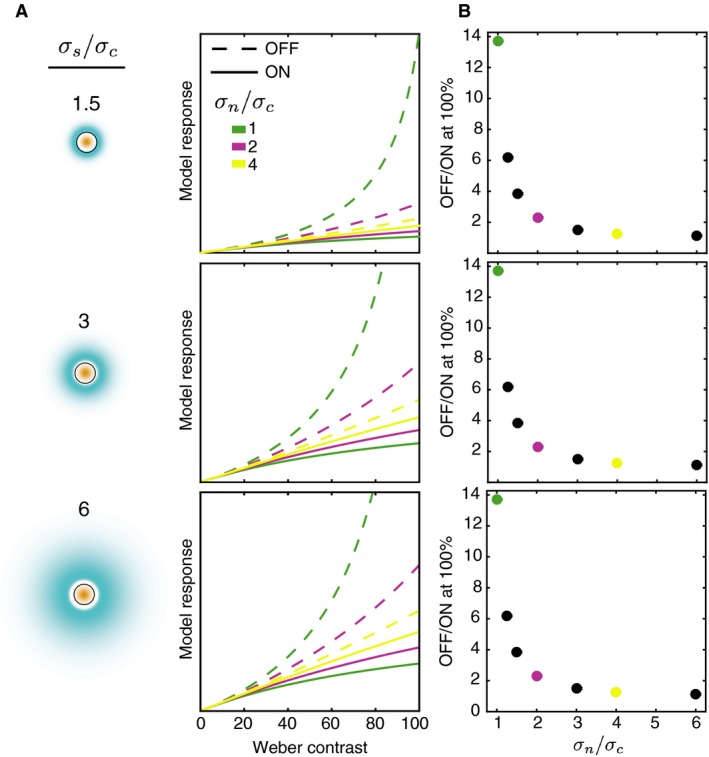
Model contrast response functions. (A) Each panel shows the contrast response function for ON and OFF units (solid and dashed lines, respectively) with a different surround/center ratio (see icons on the left). For the icons, the color map is the same as in Figure 1, but the scale differs across panels so as to make the surround visible (with large surrounds, the filter response at each point in the surround is very small). The circle outline represents the area covered by the stimulus. Within each panel, the response functions are shown for three different normalization/center ratios. The ordinate scales are matched for all panels. (B) For each of the surround/center ratios in (A), the ratio of the model OFF to ON response at 100% contrast is shown. Each point shows the results for one normalization/center ratio (abscissa). Ratios for which full response functions are shown in (A) are color coded.

Clearly, the nonlinearity and asymmetry depend on the parameters of normalization. Figure 2A shows the contrast response functions for a sample of the model units with a range of parameters. Different surround/center ratios (*σ*_*s*_/*σ*_*c*_) are shown in each panel (from top to bottom: 1.5, 3, and 6). Icons show the relative extent of the center and surround, with the size of the disk stimulus outlined in black. Within the panels, different normalization/center ratios (*σ*_*n*_/*σ*_*c*_) are shown (1, 2, and 4). Several patterns are clear. First, all model units show the same general results as in Figure 1: a greater OFF nonlinearity and a greater OFF response overall. Second, larger surround/center ratios result in larger responses overall. This is because as the antagonistic surround becomes more diffuse, the positive lobe of the DoG extends from partial to full coverage of the stimulus disk, increasing the response magnitude overall. Third, smaller adaptation/center ratios result in larger differences between the ON and OFF response functions.

Figure 2B shows the ratio of the OFF to ON responses at maximum contrast (100%) for each unit. These values are the same for each surround/center ratio (three panels), but vary as a function of the normalization/center ratio (abscissa values). Smaller normalization areas result in larger OFF/ON ratios. A given normalization area can be thought of as determining the relative weight given to the luminance of the disk and the luminance of the background in the divisor of equation (1). When the normalization area is small, the divisor is mostly determined by the disk luminance, resulting in more asymmetric responses for bright and dark disks. When the normalization area is large, more weight is given to the background, which is the same for all disks. Consistent with measurements from early visual neurons, across normalization areas the OFF/ON ratios at 100% contrast are all greater than 1 (OFF > ON). This is because, regardless of the size of the normalization area, the normalization value for a 100% contrast decrement is always lower than for a 100% contrast increment on the same background. Note that this would not be the case if the central region where the disc is present was excluded from the normalization area. In the current model, the OFF/ON ratio at 100% contrast ranges from 1.1 to 13.7, so some values are substantially larger than those reported for early visual neurons (e.g., Zaghloul et al. 2003; Kremkow et al. 2014). The very large ratio for *σ*_*n*_/*σ*_*c*_ = 1 is affected by the fact that the 100% contrast dark disk in the stimulus actually has an intensity value of 0, something that is not typically the case on physical display systems.

### Comparison to contrast response nonlinearities of retinal ganglion cells

**Figure 3:**
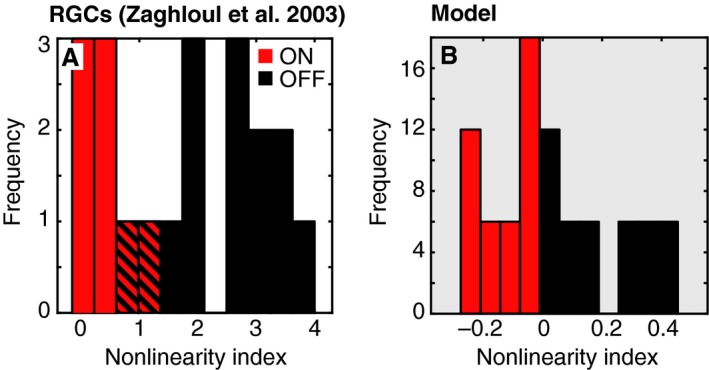
ON and OFF nonlinearities of retinal ganglion cells (RGCs) and model units. (A) Nonlinearity indices from a population of RGCs are replotted from Zaghloul et al. (2003). Striped lines indicate overlapping frequencies for ON and OFF cells. (B) Nonlinearity indices are shown for a population of model units. For clarity, panels in this figure and Figure 4 with model data have a shaded background.

We next compared the nonlinearities of all of the unique model units to nonlinearities that have been previously reported from a set of ON and OFF RGCs in the guinea pig retina (Zaghloul et al. 2003). Figure 3A–B show histograms of nonlinearity indices computed for the retinal and model data, respectively. In both cases, the indices are systematically larger for the OFF units, indicating a more accelerating nonlinearity. As it was not possible to exactly match the nonlinearity index measure between the experimental and model stimuli, these indices were also computed using several different points along the model contrast response curve. The same pattern of results was present regardless of the points used to compute the model index.

Note, however, that the range of the indices differs between the models and the RGCs: the RGCs (both ON and OFF) have a more accelerating nonlinearity than predicted by this model. ON and OFF nonlinearities reported in the literature vary, but tend to be larger than those measured in the model. Chichilnisky and Kalmar (2002) reported average OFF and ON indices of 1.1 and 0.1 in the primate retina, and Kastner and Baccus (2013) reported average OFF and ON indices of 2.2 and 1.3 in the tiger salamander retina. Thus, while the greater OFF than ON nonlinearity can be qualitatively captured by divisive normalization, additional mechanisms may serve to modify or exaggerate this difference.

### Comparison to luminance responses in the lateral geniculate nucleus

**Figure 4:**
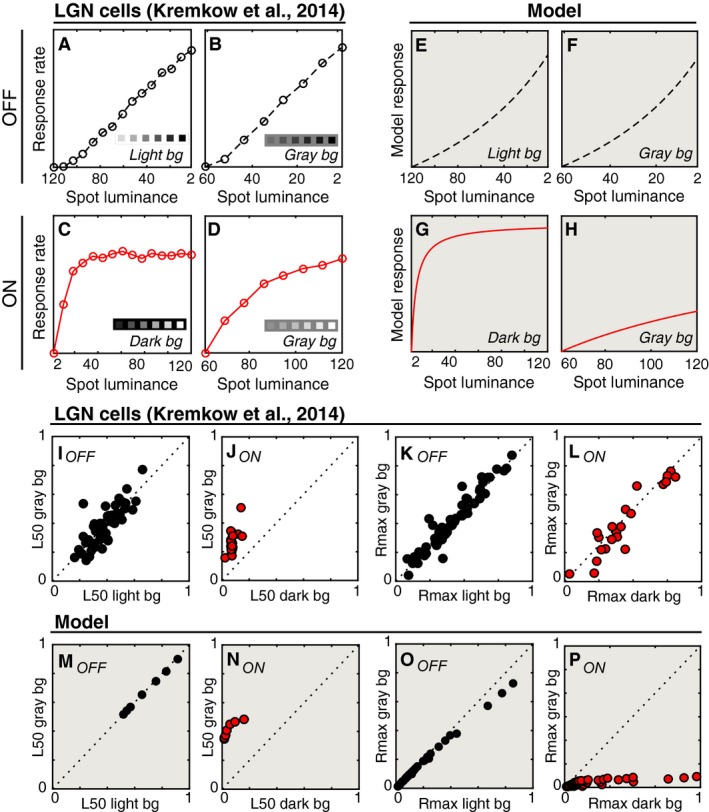
Comparison between lateral geniculate nucleus (LGN) cells and model units. (A–D) Firing rates (baselined) are shown for example OFF and ON LGN cells from Kremkow et al. (2014). The ordinate scale is the same for each panel. (A, B) show responses of an OFF cell with different luminance levels (cd/m^2^) on the abscissa. Note that the abscissa is reversed in these panels. (A) shows luminance responses on a light (120 cd/m^2^) background and (B) shows responses to a subset of the same luminances when presented on a gray (61 cd/m^2^) background (see insets). (C, D) are similar to (A, B), except firings rates are shown for an ON cell. In this case, the background luminance was either dark (C; 2 cd/m^2^) or gray (D; 61 cd/m^2^). (E–H) The responses of one example model unit are shown as in panels (A–D). (I, J) The relative luminance values at which individual LGN cells reached half of their maximum response on each background are shown as scatter plots for OFF (I) and ON units (J). (K, L) The normalized responses to the maximum contrast are shown as in panels (I, J). (M–P) Same as (I–L) except for the model population. For clarity, panels with model data have a shaded background.

A recent report characterized the response properties of ON and OFF LGN neurons in terms of their luminance response functions (Kremkow et al. 2014). This function differs only slightly from traditional contrast response measurements – the abscissa on which the spike rates are plotted is in terms of stimulus luminance against a particular background luminance, rather than contrast. The authors reported that OFF luminance responses increased with approximately the same slope regardless of the brightness of the surrounding background (see example unit replotted in Fig. 4A–B). In comparison, the ON responses on a dark background were much more compressive than the response to the same stimulus on a gray background (Fig. 4C–D).

It is not immediately obvious whether this constellation of responses in the LGN suggests different properties than those typically measured in the retina, and those captured by the current model. Thus, we compared these data to our same model. The results for a model example unit are shown in Figure 3E–H (*σ*_*s*_ = 3*σ*_*c*_ and *σ*_*n*_ = 2*σ*_*c*_). Several features of this unit are reflective of the LGN cells: the luminance responses for dark spots are similar regardless of the background level (Fig. 3E–F) and the ON responses are more compressive on the dark background (Fig. 3G–H).

A population‐level comparison revealed several similarities, and one notable difference between the LGN and model populations. In the LGN population, the relative luminance at half‐response saturation (L50) was similar for OFF cells on both backgrounds (light/gray background = 0.96), but tended to be much smaller for ON cells on a dark background (dark/gray background = 0.26) (Fig. 3I–J). These ratios were similar to the model: light/gray = 1.0, dark/gray = 0.13 (Fig. 3M–N). The model closely captured the asymmetric effects of background light level on the LGN responses.

However, in the LGN population, the responses at maximum contrast (Rmax) for both ON and OFF cells were unaffected by background luminance (OFF light/gray background = 0.96, ON dark/gray background = 1.13) (Fig. 3K–L). The model predictions for OFF units were in agreement with the LGN OFF cells (light/gray background = 1.06), but the predictions for ON units differed substantially (dark/gray background = 4.90) (Fig. 3O–P). The reason for this mismatch is clear: the model will always respond more to greater contrast levels, within a given polarity. So for ON units, the model predicts a much larger response to a given luminance if it appears on the darker background. However, the response of the LGN cells to 120 cd/m^2^ is very similar on both a dark and a gray background (Fig. 4C and D).

Thus, while their nonlinearities are well‐captured by the current model, clearly the LGN responses are modulated by absolute luminance in a way that does not reflect *within polarity* contrast constancy. To fully explain these data, an additional mechanism that selectively boosts ON responses is necessary. Interestingly, this runs contrary to the common assessment that early visual processing favors darks over brights (Ratliff et al. 2010; Kremkow et al. 2014).

## Discussion

This report shows that a simple contrast‐encoding model that has been proposed in the literature shares several bright/dark response asymmetries with precortical visual processing. These similarities arise without ever directly fitting the model to a specific dataset, and the same model predicts features from different species, different cell types, and different recording preparations.

### Other ON/OFF asymmetries

Obviously, there are a range of asymmetries between the ON and OFF pathways that cannot be accounted for by the current model. In addition to those highlighted in the Results, these include the higher baseline firing rate of ON cells (and the ability of ON cells to signal decrements), the fact that OFF RGCs are more numerous, and differences in temporal dynamics and receptive fields (Dacey and Petersen 1992; Benardete and Kaplan 1999; Chichilnisky and Kalmar 2002; Zaghloul et al. 2003; Pandarinath et al. 2010; Ratliff et al. 2010; Jin et al. 2011; Nichols et al. 2013). Although, with regards to relative numerosity, it remains unclear if this asymmetry exists across all species (Baden et al. 2016), and with regards to temporal asymmetries, other studies have not found compelling asymmetries between ON and OFF neuronal responses (Kremers et al. 1993; Benardete and Kaplan 1997). In addition, across the many species that have been studied, other types of RGCs transmit contrast information, such as ON‐OFF RGC‐types that respond to both light increments and decrements (Masland 2012; Baden et al. 2016). Examining the role of these properties of early visual processing will further our understanding of how visual information is represented. New resources that allow for the detailed prediction and exploration of retinal responses promise to expand our ability to examine a range of physiologically plausible early visual models (Wohrer and Kornprobst 2009; ISETbio 2015).

### Normalization mechanisms

In the current model, the receptive field is spatially antagonistic, but the normalization area is not. While the current model is a conceptual encoding model for spatial contrast, rather than a mechanistic model of early visual processing, this form of normalization may be consistent with retinal adaptation mechanisms that commence prior to the formation of antagonistic receptive fields. Here, we will examine similarities between the current model and photoreceptor adaptation. Indeed, origins of ON/OFF contrast response asymmetries at the level of the photoreceptors have been proposed previously (Angueyra and Rieke 2013; Baden et al. 2013) and explicitly modeled (Kremkow et al. 2014; Carandini 2016). Other asymmetries between ON and OFF neurons (different spatial receptive field properties) have similarly been suggested to derive from photoreceptor responses (Kremkow et al. 2014).

Measurements from cone photoreceptors that have been adapted to a steady light level show that these cells hyperpolarize in response to increases in light intensity and depolarize in response to decreases in light intensity – relative to a baseline resting potential (Baylor and Fuortes 1970; Normann and Perlman 1979). The normalized photoreceptor polarization (*V*) in response to a given light intensity (*I*_*b*_) can be generally described by the following equation:

(3) $$V=\frac{I_{b}}{I_{b}+I_{a}},$$

where *I*_*a*_ is the stimulus intensity that produces half of the maximum response. Previous work has shown that the magnitude of *I*_*a*_ advances systematically when photoreceptors are adapted to greater light levels such that the dynamic range of cone responses is roughly centered around the adapted intensity level (Boynton and Whitten 1970; Normann and Perlman 1979).

**Figure 5:**
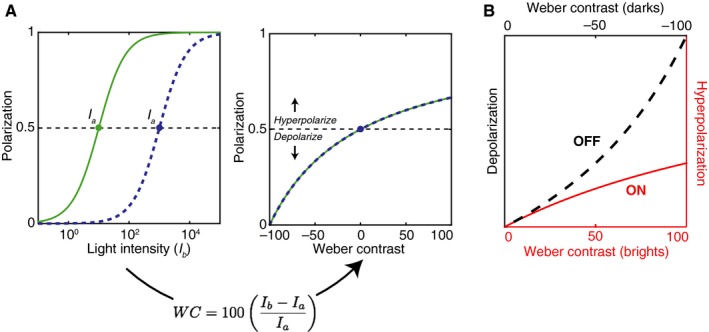
Contrast asymmetry in cone polarization. (A) In the left panel, intensity response functions are shown for a model cone photoreceptor adapted to two different light intensities (*I*_*a*_). The horizontal dashed line represents the baseline polarization. In the right panel, the same data are plotted with the abscissa converted to Weber contrast. (B) Hyperpolarization and depolarization responses are overlaid to illustrate the different nonlinearities providing input to OFF bipolar cells for dark contrasts, and ON bipolar cells for bright contrasts.

Figure 5A shows predicted cone response curves for two different adaptation levels based on this equation. When plotted in terms of light intensity on a log axis (left panel), the depolarizing and hyperpolarizing responses are symmetric and centered around the adapted value (*I*_a_). When the light intensity levels are converted to Weber contrast (right panel), it is clear that the depolarization in response to dark contrasts (negative values) is more accelerating than the hyperpolarization in response to bright contrasts (positive values). Note that in this case, Weber contrast represents the contrast *over time* rather than space (i.e., how much brighter or darker was *I*_*b*_ relative to the previously adapted light level *I*_*a*_). The ON and OFF bipolar cells that form the initial separation into ON and OFF pathways either preserve this polarization (OFF) or invert it (ON). To illustrate this, Figure 5B superimposes the hyperpolarization and depolarization curves from 5A for positive (red line) and negative (black line) Weber contrasts. The shapes of these curves are extremely similar to those produced by the current model (See Fig. 2).

The similarity derives from the fact that these curves are produced by a normalization factor that includes the sum of the currently adapted light intensity and a second brighter or darker light level (eq. (3)). This is similar to the Gaussian normalization used in the current model (eqs. (1) and (2)), which averages over both the central stimulus and surround areas. Thus, these cone responses share the general features that produce ON/OFF asymmetries for spatial contrast in the model, but for temporal contrast: constancy within polarities, but not between them. At present, these effects are difficult to directly relate because, for example, in the Kremkow et al. (2014) study, the background luminance that preceded the onset of bright and dark stimuli was the same as the luminance of the surrounding area, thus temporal and spatial contrast were correlated. However, if one wanted to predict early visual responses to natural images, it may be reasonable to use the current model and assume that local spatial and temporal contrast are correlated in these signals as well (Ratliff et al. 2010; Cooper and Norcia 2015).

It is also notable that several studies have reported that OFF pathway cells are actually less sensitive to low contrasts than ON cells (Chichilnisky and Kalmar 2002; Zaghloul et al. 2003). This is contrary to the prediction of a normalization‐driven process, because in this model the slope and magnitude of the OFF contrast response function will be greater than the ON slope at all contrasts.

### Cortical asymmetries

Characterizing the principles that govern early visual responses is essential for understanding retinal processing, but it will also be crucial for interpreting a spate of recent results showing downstream cortical bright/dark asymmetries (Yeh et al. 2009; Xing et al. 2010; Polack and Contreras 2012; Kremkow et al. 2014; Liu and Yao 2014; Veit et al. 2014; Zurawel et al. 2014; Tan et al. 2015). Cortical asymmetries may reflect an extension of the asymmetries explored here, or may reflect additional optimizations for patterns in natural images (Ratliff et al. 2010; Tkacik et al. 2010; Clark et al. 2014; Cooper and Norcia 2015). To differentiate these possibilities, rather than model cortical asymmetries via explicitly different ON/OFF mechanisms (Liu and Yao 2014; Zurawel et al. 2014), it may be valuable to consider to what extent normalization models can and cannot also predict these downstream effects. This could provide further insight into cortical representations of visual contrast at the level of neuronal populations (Naselaris and Kay 2015). Similarly, normalization has been proposed to play a role in perceptual asymmetries between brights and darks, which generally reflect greater sensitivity to darks (Chubb and Nam 2000; Lu and Sperling 2012; Haun and Peli 2013).

## Conclusion

A simple description of the ingredients for visual contrast responses is a high priority for understanding visual encoding. We propose that a symmetric ON/OFF model that incorporates divisive normalization can provide a simple and useful starting point.

## Conflict of Interest

The author declares no competing financial interests.
